# Game Analysis of Olympic, World and European Championships in Men’s Handball

**DOI:** 10.2478/v10078-012-0084-7

**Published:** 2012-12-30

**Authors:** Murat Bilge

**Affiliations:** 1 Kırıkkale University, School of Physical Education and Sport

**Keywords:** Handball, Position Efficiency, Technical Game Analysis

## Abstract

The development of men’s handball was analyzed using data from the Olympic, World and European Championships held within the last eight years. The most obvious change, especially within these last nine tournaments, was that men’s handball was played more dynamically and rapidly, both in attack and defense, especially by European teams. The first aim of this study was to conduct a technical analysis of current handball and to determine factors related to success in this sport discipline. The second aim was to compare the data of European Championships with other tournaments, considering the success of European teams in Men’s World Handball. The technical variables used to compare the tournaments included: the average number of attacks, the efficiency of attacks, the efficiency of goal throws, fast break goals per game, the efficiency of fast breaks, the efficiency of the goalkeeper, saves by the goalkeeper per game, number of turnovers per game, and the efficiency of position throws (wing, pivot, back court, break-through, fast break, and 7-meter). This technical analysis used cumulative statistics from the European Handball Federation and International Handball Federation. ANOVA revealed significant differences between the first eight teams in the European Championships and their counterparts in the other two tournaments (Olympics and World Championships) in terms of several technical variables. The results showed that the efficiency of fast break, pivot position and back court players affected the ranking in favor of the European teams in significant international tournaments.

## Introduction

Performance measurement is one of the main subjects of movement and training sciences. Game analysis methods used in this field have gradually improved. Many of the most popular and original recent studies in this field have involved recording performance variables during or after competitions, and visual and written storage of these records in the computer environment. The measurement and assessment of performance play an important role in planning the training process and competition (Taborsky, 2011).

Game analyses aim to determine the individual performances of athletes, to evaluate general conditioning, technical and tactical skills of the teams, and to assess the general and individual performances of opposing teams (Vuleta, 2007). Analyses are the key factors of general audit and periodization assessment, which could shape the scope of training programs (König, 2010).

Post-game analyses aim to assess performance of a team in that game, while mass analyses following a specific season, tournament or championship present the success or failure of the teams, and even the general assessment of the branch itself (Pollany, 2006).

The study by Sevim and Bilge (2007) showed that, due to changes in the rules of the game, rapid re-starts following a goal and, especially, interpretations made by the rule of passive game resulted in top teams to pay more attention to fast break strategies, and made them sustain this rapid game during the attack position. The preparation period of the attack position was reduced, thus bringing out a dynamic and rapid game, and resulting in richer and more sophisticated forms of strategy (Pokrajac, 2010).

The Olympics, World Championships and European Championships are tournaments where top-level performances occur for a certain sports branch. Comparison of the tournament analyses and longitudinal analysis of matches during these tournaments are of primary importance to determine current developments in world handball (Taborsky, 2007). From this point of view, this study aims to examine the statistics of the teams ranked among the top eight in men’s handball competitions during the Olympics, World Championships and European Championships which were held within the last eight years, and to assess these statistics according to the tournaments and years. Another aim of this study is to analyze target matches in the European Championships, as these teams constitute 92.5% of the European men’s teams ranked in the Olympics and World Championships, which are the most important handball tournaments.

## Material and Methods

The study includes total team statistics of the teams ranked among the top eight in men’s handball competitions during the 2004–2008 Olympics, 2005–2007–2009 World Championships, and 2004–2006–2008–2010 European Championships. Official competition statistics from the European and International Handball Federations were used. Both the IHF and EHF use the same game analysis process as the method of data collection. This study was presented in the 11^th^ International Sport Sciences Congress, held in 10–12 November 2010, Antalya, Turkey.

At first, the study examined the average number of attacks, attack efficiency, shot efficiency, average fast break goals per game, fast break efficiency, goalkeeper efficiency, average goalkeeper saves per game, average number of exposures to foul per game, and differences in the ratios of position throws (wing, pivot, back court, break-through, fast break, and 7-meter) to all goals (Hergeirsson, 2008).

In the second phase, the same variables were compared by taking the Olympics and World Championships as one group, and the European Championships as another.

### Statistical Analysis

The technical variables were examined using descriptive statistics.

The Kruskal-Wallis test was used to determine any differences between technical parameters. In case of differences between groups, the Scheffe Post-Hoc test was used to determine from which tournament such differences arose.

The T-test was used for independent samples regarding the variety of technical parameters obtained from the tournaments of different classifications.

## Results

The present researcher took into consideration success in tournaments, and thus focused on the top eight teams. In the total analyses, the most important quantitative variable is the number of games. Therefore, to standardize comparison between the teams, an equal number of games have to be considered. In these tournaments, every game is important, and all of the top-eight teams reached the end of these tournaments. In this study, the opponent’s position was ignored.

Table 1 shows the descriptive statistics of the related variables obtained from the nine tournaments examined.

In terms of the number of attacks, there was no statistical difference between the tournaments (X^2^=11.250, p>0.05). In other words, there was a similar number of attacks in different tournaments.

In terms of attack efficiency, the 2004 Olympics differed significantly from the 2006 European Championship and 2007 World Championship (X^2^=23.482, p<0.05, Table 2).

In terms of shot efficiency, there was no statistical difference between the tournaments (X^2^=16.788, p>0.05). In other words, shot efficiency variables were similar in different tournaments.

In terms of fast break goals per game, there was a statistical difference between the 2004 Olympics and the 2010 European Championship; and between the 2004 and 2010 European Championships and the 2005 – 2007 – 2009 World Championships (X^2^=39.734, p<0.01, Table 3).

In terms of fast break efficiency, there was a statistical difference between the 2004 Olympics and 2008 European Championship and between the 2008 European Championship and 2010 European Championship (X^2^=28.823, p<0.01, Table 4).

In terms of goalkeeper efficiency, there was no statistical difference between the tournaments (X^2^=8.159, p>0.05). In other words, goalkeeper efficiency variables were similar in all of the tournaments examined.

In terms of goalkeeper saves per game, there was no statistical difference between the tournaments (X^2^=4.897, p>0.05). The number of goalkeeper saves per game was similar in the analyzed tournaments.

There was no statistical difference between the tournaments in terms of the number of exposures to fouls per game (X^2^=6.903, p>0.05). The number of turnovers per game was similar in the nine world handball events.

There was no statistical difference between the tournaments in terms of the ratio of wing position goals to all goals (X^2^=11.296, p>0.05). There was a similar number of wing position goals in all tournaments.

In terms of the ratio of pivot position goals to all goals, the 2008 European Championship was significantly different from the 2004 Olympics and 2005 World Championship (X^2^=27.333, p<0.01, Table 5).

Considering the ratio of back court position goals to all goals, the 2005 World Championship was significantly different from the 2008 European Championship (X^2^=24.367, p<0.01, Table 6).

In terms of the ratio of break-through position goals to all goals, there was no statistical difference between the tournaments (X^2^=11.121, p>0.05). There was a similar number of breakthrough position goals in all tournaments analyzed.

The ratio of fast break goals to all goals was significantly different in the 2008 Olympics compared with the 2010 European Championship (X^2^=20.073, p<0.05, Table 7).

There was no statistical difference between the tournaments for the ratio of 7-meter goals to all goals, (X^2^=10.988, p>0.05). There was a similar number of 7-meter goals scored in different tournaments.

Of the fifteen statistically significant differences found during the comparisons above, thirteen were between European and other Championships, and only 7.5% (3/40) of the top-eight ranked teams in the Olympic and World Championships were from outside Europe. This finding led us to analyze the European data in comparison to other international championships (Table 8).

T-tests of particular technical variables obtained from the tournaments of different classifications showed that:

- Average number of fast break goals per game was higher in Olympic Games and World Championships than in the European Championships.
- Fast break efficiency rates were higher in Olympic Games and World Championships than in the European Championships.
- The ratio of pivot position goals to all goals in Olympic Games and World Championships was higher than in European Championships.
- The ratio of back court position goals to all goals in Olympic Games and World Championships was lower than in the European Championships.
- The ratio of break-through goals to all goals in Olympic Games and World Championships was lower than in European Championships.
- The ratio of fast break goals to all goals in Olympic Games and World Championships was higher than in European Championships.

## Discussion

In team sports, the interpretation of game performance has motivated researchers and coaches to develop tactical indicators associated with sports success (Massuça, 2009). In handball, as in basketball, fast break efficiency is the main factor determining success among teams of the same level (Yang, 2006; Fernandez, 2009).

Creating a model of a handball game is a priority of the epistemological investigative work of coaches (Alexandru, 2009). Based on the causal model of performance, it was stated that handball performance results from the complex capacity of combining a set of capabilities (e.g., mental, physiological, technical). These capacities create different and complex actions to solve the problems throughout the game, and within this context, they are essential to present a balanced domain of the factors that influence sports performance (e.g., morphological, physical, technical, tactical and psychosocial) (Massuça 2009). This study examined the statistics of the top-eight ranked men’s handball teams from the Olympics, World Championships and European Championships that were held within the last eight years; and assessed these data according to the tournaments and years. All tournaments examined in the study were played under the rules changed in 2000.

In terms of fast break efficiency, which is the key element of modern handball, European teams had fewer opportunities for fast breaks in competitions among themselves, while they had the increased number and effectiveness of fast break actions against their non-European opponents in the Olympics and World Championships. This was the most important advantage of European teams, from both strategic and physical perspective (Johansson, 2004; Pokrajac, 2010).

The fast break has become one of the main concerns for all good teams and also an efficient way of scoring goals. Beginning with analysis of games played in the European and World Championships and Olympics, we synthesize the main aspects of the modern game. A fast break represents the phase in which the ball, reentered in the possession of the defending team in the shortest way (goalkeeper – player or goalkeeper – intermediary – player), is finalized and the opponent players do not have sufficient time to position themselves in an organized system of defense. Due to its efficiency, the fast break should be used by every team aiming to succeed at the highest levels of the sport (Calin, 2010).

Calin (2010) studied fast break efficiency on the top-four teams at the World Championship in China, 2009, and found that the place of the fast break in modern female handball was extremely precise, and accounted for 23% (1351) of all goals scored at that championship.

Based on cumulative statistics from the 2009 World Championships, Alexandru (2009) found that the most effective shooting position for scoring was the fast break throws (88.23%), followed by break-through position shots (75%). Analysis of the contribution of goal situations for scoring showed that pivot position efficiency was the second most efficient, at the rate of 15.63%.

Yiannakos et al. (2005) categorized attacks from fifteen matches of eight teams participating in the first division of the 2003 National Men's Handball Championship. They reported a significant difference between the two halves of each match regarding the effectiveness of the fast break.

Srhoj et al. (2001) analyzed the influence of eighteen predictive variables on the outcomes of eighty top level handball games, to establish the significance of positional direction of the attack end on successful plays. The frequency and the effectiveness of shooting from different playing positions were defined by these predictive variables. They reported that the pivot attacker position, the break-through and fast break shoots had significant influences on resulting success (Srhoj et al., 2001).

Situational efficiency of players, or of a team, can be observed in different phases and subphases of play during a match. The main phases of handball play are attack and defense, depending on ball possession. Two transitional phases, the phase of returning to defense and the phase of a counter-attack, are derived from the main phases (Gruiç, 1997).

Gruiç et al. (2006) investigated situation-related efficiency or performance using a sample of 60 handball matches. Twenty-four different national teams were divided into four preliminary groups of six teams. The criterion variable was defined as the final match outcome. They reported that the characteristics of successful teams with better fast break scoring efficiency were as follows: adequate defense system selection, quick reaction to the opponent‘s unsuccessful shot, fast running (by sprint and by “sharp” and accurate ball transmission), and a good selection of shooting techniques.

In women’s handball the same was true for fast break efficiency. Ohnjec et al. (2003) reported that fast break goals had a significant positive influence on goal-difference during the 2003 women’s World Championships in Croatia. The successful teams used the fast break to score “easy goals” more often than losing teams.

According to the statistics of the 2007 World Women Handball Championship and the Beijing Olympic Women Handball Tournaments in terms of technique and tactics, Jian (2011) reported that top teams had common characteristics, including effective fast breaks (efficiency of 70%, 7.1 points each game) of the China team.

In terms of a national handball league, Oscar et al. (2011) studied the performance of players according to specific positions in the ASOBAL league 2008–2009. The sample group comprised 192 players, of which 27 were left wing, 38 left back, 35 centre back, 25 right back, 30 right wing and 37 pivots. Fouls were recorded in official statistics of the 240 games that season and a descriptive statistical analysis was performed. The results clearly show how the first line makes the most 9-meter shots, the most assists, technical fouls, regulation fouls and lost balls. Meanwhile, the wings predominate in fast break goals and pivots in 6-meter actions. On the other hand, wings and backs scored the most goals in the most remote areas of the goal, while the centre back and back made more shots to the right of the goal.

In the same national league, Gutierrez et al. (2011) used discriminate analysis to examine differences between winners and losers in the 2008–2009 season. They reported that winning teams achieved a higher average number of goals from all throwing distances. However, the greatest differences between winners and losers were observed in fast break goals and shots. These were the only two throwing statistics that showed significant differences between successful and unsuccessful teams.

Another important factor is related to playing positions. European teams used more pivot and break-through positions than back court positions, which are considered as distant throws and less effective than other positions. This shows that European teams made more strategic preparation regarding the emphasis on close-range throws and throw-ins (Pollany, 2006; Sevim, 2006; Spate, 2004).

Gabriel (2011) found that the importance of pivot skills increased in a game between equally matched teams.

Meletakos et al. (2011) assessed the relative importance of selected performance indicators in modern top-level handball through the analysis of offensive actions in three consecutive men's world championships (2005, 2007 and 2009). Their results demonstrated a strong relationship between six-meter and nine-meter offensive actions, as evidenced by their very high negative correlation coefficients in both the throw attempts and goals scored. Interestingly, the nine-meter efficacy remained relatively constant throughout the three competition years, while the six-meter efficacy showed a significant increase in competition years 2007 and 2009 compared to 2005, as a result of the appearance of highly qualified top ranking players in the pivot position.

In conclusion, European teams have superiority in men’s handball over other teams (Sevim and Bilge, 2007). The technical variables contributing to this superiority show that the fast break, pivot position and back court position efficiencies of the European teams are more evident in Olympics and World Championships.

New trends in technical, strategic and fitness development of handball require new technical and motor characteristics of handball players. Handball increasingly requires players to be quicker, more dynamic, versatile in both attack and defense, technically qualified, able to play at each position at least for a short time and to have excellent game perception (Pokrajac, 2007; Taborsky, 2008).

During this study, European teams were ranked in the top eleven in the last World Championship held in Switzerland in January 2011, demonstrating the validity of this study.

In order that such cumulative analyses could be effective in the future, they should provide the coaches and the players with feedback. Considering the findings of the present research, significant statistical differences leading the teams to success are based on the fast break tactics and throwing preferences of the teams in the set offense. Coaches should plan not only tactical training programs but also programs considering the physical, physiological and psychological strength of the players that can endure such an intensive tempo in order to improve the efficiency of the attacks. On the other hand, preferences regarding the positions of the players should be reflected in the practice sessions. Moreover, coaches should consider these variables of success in long-term planning, and apply them in the training of youth players while they raise the elite handball players of the future.

## Figures and Tables

**Table 1:** General Descriptive Statistics of Top-Eight Ranked Teams in 2 Olympics, 3 World Championships and 4 European Championships

Technical Parameters (n=72)	M	SD	Min	Max
Number of attacks	57.55	2.56	49.6	65.0
Attack Efficiency (%)	50.86	2.84	41	58
Shot Efficiency (%)	57.65	3.21	49	67
Fast Break Goals Per Game	5.71	1.78	2.37	10.00
Fast Break Efficiency (%)	73.00	8.94	47	96
Goalkeeper Efficiency (%)	34.19	3.04	26	41
Goalkeeper Saves Per Game	13.91	1.52	10.28	17.12
Number of Exposure to Faults Per Game	12.11	1.66	8.75	17.62
The Ratio of Wing Position Goals to All Goals	13.73	3.34	5.6	25.8
The Ratio of Pivot Position Goals to All Goals	19.39	4.70	9.9	35.2
The Ratio of Back Court Position Goals to All Goals	30.05	6.35	18	48
The Ratio of Break-through Goals to All Goals	9.56	3.67	4.0	20.9
The Ratio of Fast Break Goals to All Goals	16.98	3.91	9.2	26.0
The Ratio of 7-meter Goals to All Goals	10.27	2.84	5.0	16.8

**Table 2:** Kruskal-Wallis Analysis of Attack Efficiency (%) of Teams

	M	SD	x^2^	p	Statistical Differences
2004 Olympics	47.38	3.37	23.482	.003	Oly 2004-ECh 2006 Oly 2004-WCh 2007
2004 European Championship	50.38	1.68			
2005 World Championship	51.00	4.00			
2006 European Championship	53.13	2.03			
2007 World Championship	52.88	1.24			
2008 Olympics	50.38	2.44			
2008 World Championship	50.13	1.24			
2009 World Championship	51.38	2.61			
2010 World Championship	51.13	2.41			

**Table 3:** Kruskal-Wallis Test Results of Average Fast Break Goals Per Game

	M	SD	x^2^	p	Statistical Differences
2004 Olympics	6.74	1.72	39.734	.000	Oly 2004 – ECh 2010 ECh 2004 – WCh 2005 ECh 2004 – WCh 2007 ECh 2004 – WCh 2009 ECh 2010 - WCh 2005 ECh 2010 - WCh 2007 ECh 2010 - WCh 2009
2004 European Championship	4.20	.98			
2005 World Championship	7.18	1.50			
2006 European Championship	4.79	1.10			
2007 World Championship	7.06	1.18			
2008 Olympics	5.63	1.25			
2008 World Championship	4.89	.72			
2009 World Championship	7.24	1.51			
2010 World Championship	3.66	1.24			

**Table 4:** Kruskal-Wallis Test Results for Fast Break Efficiency of the Teams

	M	SD	x^2^	p	Statistical Differences
2004 Olympics	78.63	7.67	28.823	.000	Oly 2004 – ECh 2008 ECh 2008 – ECh 2010
2004 European Championship	64.50	4.59			
2005 World Championship	75.75	7.83			
2006 European Championship	72.75	6.86			
2007 World Championship	76.13	3.90			
2008 Olympics	72.38	6.23			
2008 World Championship	62.38	7.55			
2009 World Championship	76.25	5.03			
2010 World Championship	78.25	13.18			

**Table 5:** Kruskal-Wallis Test Results Regarding the Ratio of Pivot Position Goals of the Teams to All Goals

	M	SD	x^2^	p	Statistical Differences
2004 Olympics	22.20	3.68	27.333	.001	Oly 2004 – ECh 2008 WCh 2005 – ECh 2008
2004 European Championship	18.56	3.56			
2005 World Championship	23.35	6.84			
2006 European Championship	17.78	4.07			
2007 World Championship	22.04	3.00			
2008 Olympics	21.05	3.92			
2008 World Championship	14.07	2.71			
2009 World Championship	18.81	3.45			
2010 World Championship	16.70	2.84			

**Table 6:** Kruskal-Wallis Test Results Regarding the Ratio of Back Court Position Goals of the Teams to All Goals

	M	SD	x^2^	p	Statistical Difference
2004 Olympics	27.31	3.98	24.367	.002	WCh 2005 – ECh 2008
2004 European Championship	28.55	4.57			
2005 World Championship	23.01	4.71			
2006 European Championship	32.02	2.94			
2007 World Championship	31.07	6.33			
2008 Olympics	27.92	9.90			
2008 World Championship	35.66	4.35			
2009 World Championship	33.07	5.63			
2010 World Championship	31.87	4.93			

**Table 7:** Kruskal-Wallis Test Results Regarding the Ratio of Fast Break Goals of the Teams to All Goals

	M	SD	x^2^	p	Statistical Difference
2004 Olympics	19.10	3.96	20.073	.010	Oly 2008 – ECh 2010
2004 European Championship	14.42	3.08			
2005 World Championship	18.09	4.19			
2006 European Championship	15.44	3.29			
2007 World Championship	17.36	2.89			
2008 Olympics	19.89	3.84			
2008 World Championship	17.41	2.77			
2009 World Championship	18.37	2.54			
2010 World Championship	12.69	3.79			

**Table 8:** T-tests of the Different Technical Parameters Obtained From the Tournaments of Different Classifications

	Olimpics and World Ch. (n=40)		European Ch. (n=32)		

	M	SD	M	SD	t
Number of attack	57.95	2.54	57.06	2.54	1.469
Attack Efficiency (%)	50.60	3.29	51.19	2.16	−.869
Shot Efficiency (%)	57.75	3.69	57.53	2.54	.285
Fast Break Goals Per Game	6.77	1.49	4.38	1.10	7.523**
Fast Break Efficiency (%)	75.83	6.32	69.47	10.47	3.185**
Goalkeeper Efficiency (%)	34.73	2.71	33.53	3.34	1.675
Goalkeeper Saves Per Game	14.00	1.31	13.80	1.76	3.185
Number of Exposure to Faults Per Game	12.27	1.79	11.91	1.49	.918
The Ratio of Wing Position Goals to All Goals	13.11	3.42	14.51	3.13	−1.780
The Ratio of Pivot Position Goals to All Goals	21.49	4.44	16.78	3.61	4.846**
The Ratio of Back Court Position Goals to All Goals	28.48	7.03	32.03	4.79	−2.435*
The Ratio of Break-through Goals to All Goals	8.58	2.99	10.781	4.11	−2.620*
The Ratio of Fast Break Goals to All Goals	18.56	2.99	14.99	4.11	4.291**
The Ratio of 7-meter Goals to All Goals	9.76	2.49	10.90	3.16	−1.716

* p< ,05

** p< ,01
